# Supplementary material for: Genetic diversity of Trypanosoma cruzi parasites infecting dogs in southern Louisiana sheds light on parasite transmission cycles and serological diagnostic performance
Source: PLoS Negl Trop Dis. 2020 Dec 17;14(12):e0008932. doi: 10.1371/journal.pntd.0008932 (PMC7775123; doi:10.1371/journal.pntd.0008932)
Supplement: S1 Table — (PDF) [file pntd.0008932.s001.pdf]

Supplementary Table S1. List of mini-exon sequences from dogs.

Sequence ID	Parish	Dog ID	DTU	Accession #
BRO-77133-TcI-H1	East Baton Rouge	77133	TcI	MT365367
BRO-77133-TcI-H2	East Baton Rouge	77133	TcI	MT365335
BRO-79591-TcI-H1	East Baton Rouge	79591	TcI	MT365272
BRO-79591-TcI-H2	East Baton Rouge	79591	TcI	MT365370
CAL-1712736-TcI-H1	Calcasieu	1712736	TcI	MT365336
CAL-1712736-TcI-H2	Calcasieu	1712736	TcI	MT365301
CAL-1712736-TcI-H3	Calcasieu	1712736	TcI	MT365337
CAL-1712736-TcI-H4	Calcasieu	1712736	TcI	MT365340
CAL-1712736-TcI-H5	Calcasieu	1712736	TcI	MT365338
CAL-2212299-TcI-H1	Calcasieu	2212299	TcI	MT365342
CAL-2212299-TcI-H2	Calcasieu	2212299	TcI	MT365300
CAL-2212299-TcIV-H1	Calcasieu	2212299	TcIV	MT365414
CAL-2212299-TcIV-H2	Calcasieu	2212299	TcIV	MT365415
CAL-2229296-TcI-H1	Calcasieu	2229296	TcI	MT365359
CAL-2229296-TcI-H2	Calcasieu	2229296	TcI	MT365311
CAL-2229296-TcI-H3	Calcasieu	2229296	TcI	MT365287
CAL-2229296-TcI-H4	Calcasieu	2229296	TcI	MT365283
CAL-2229296-TcI-H5	Calcasieu	2229296	TcI	MT365319
CAL-2229296-TcI-H6	Calcasieu	2229296	TcI	MT365317
CAL-2229296-TcI-H7	Calcasieu	2229296	TcI	MT365315
CAL-2229296-TcI-H8	Calcasieu	2229296	TcI	MT365314
CAL-2261230-TcI-H1	Calcasieu	2261230	TcI	MT365353
CAL-2261230-TcIV-H1	Calcasieu	2261230	TcIV	MT365421
CAL-2263240-TcI-H1	Calcasieu	2263240	TcI	MT365270
CAL-2263240-TcI-H2	Calcasieu	2263240	TcI	MT365368
CAL-2263247-TcI-H1	Calcasieu	2263247	TcI	MT365303
CAL-2263247-TcI-H2	Calcasieu	2263247	TcI	MT365295
CAL-2263247-TcI-H3	Calcasieu	2263247	TcI	MT365293
CAL-2263247-TcI-H4	Calcasieu	2263247	TcI	MT365302
CAL-2269274-TcI-H1	Calcasieu	2269274	TcI	MT365362
CAL-2275161-TcI-H1	Calcasieu	2275161	TcI	MT365334
CAL-2275161-TcI-H2	Calcasieu	2275161	TcI	MT365345
CAL-2275851-TcI-H1	Calcasieu	2275851	TcI	MT365351
CAL-2275851-TcI-H2	Calcasieu	2275851	TcI	MT365347
CAL-2275851-TcIV-H1	Calcasieu	2275851	TcIV	MT365420
CAL-2288697-TcI-H1	Calcasieu	2288697	TcI	MT365277
CAL-2288697-TcI-H2	Calcasieu	2288697	TcI	MT365350
CAL-2288697-TcI-H3	Calcasieu	2288697	TcI	MT365320
CAL-2288697-TcI-H4	Calcasieu	2288697	TcI	MT365274
CAL-2288697-TcIV-H1	Calcasieu	2288697	TcIV	MT365422

CAL-2292546-TcI-H1	Calcasieu	2292546	TcI	MT365290
CAL-2292546-TcI-H2	Calcasieu	2292546	TcI	MT365309
CAL-2292546-TcI-H3	Calcasieu	2292546	TcI	MT365305
CAL-2292546-TcI-H4	Calcasieu	2292546	TcI	MT365291
CAL-2292546-TcI-H5	Calcasieu	2292546	TcI	MT365292
CAL-2292546-TcI-H6	Calcasieu	2292546	TcI	MT365310
CAL-2292546-TcI-H7	Calcasieu	2292546	TcI	MT365307
CAL-2292546-TcI-H8	Calcasieu	2292546	TcI	MT365308
CAL-2297800-TcI-H1	Calcasieu	2297800	TcI	MT365294
CAL-2297800-TcI-H2	Calcasieu	2297800	TcI	MT365306
CAL-2297800-TcI-H3	Calcasieu	2297800	TcI	MT365297
CAL-2297800-TcIV-H1	Calcasieu	2297800	TcIV	MT365423
DSO-9225-TcI-H1	De Soto	9225	TcI	MT365251
DSO-9264-TcI-H1	De Soto	9264	TcI	MT365261
DSO-9264-TcI-H2	De Soto	9264	TcI	MT365252
DSO-9264-TcI-H3	De Soto	9264	TcI	MT365263
DSO-9264-TcI-H5	De Soto	9264	TcI	MT365262
IBA-A33474305-TcI-H1	Iberia	A33474305	TcI	MT365364
IBA-A33474305-TcI-H2	Iberia	A33474305	TcI	MT365348
IBA-A33474305-TcI-H3	Iberia	A33474305	TcI	MT365349
IBA-A33474305-TcI-H4	Iberia	A33474305	TcI	MT365271
IBA-A33474305-TcIV-H1	Iberia	A33474305	TcIV	MT365411
IBA-A34643648-TcI-H1	Iberia	A34643648	TcI	MT365269
IBA-A34932160-TcI-H1	Iberia	A34932160	TcI	MT365326
IBA-A34932160-TcI-H2	Iberia	A34932160	TcI	MT365328
IBA-A34932160-TcI-H3	Iberia	A34932160	TcI	MT365329
IBA-A34932160-TcII-H1	Iberia	A34932160	TcII	MT365401
IBA-A34932160-TcII-H2	Iberia	A34932160	TcII	MT365396
IBA-A34932160-TcII-H3	Iberia	A34932160	TcII	MT365407
IBA-A34932160-TcII-H4	Iberia	A34932160	TcII	MT365397
IBA-A34932160-TcII-H5	Iberia	A34932160	TcII	MT365395
IBA-A34932160-TcII-H6	Iberia	A34932160	TcII	MT365398
IBA-A34932160-TcII-H7	Iberia	A34932160	TcII	MT365404
IBA-A34932160-TcVI-H1	Iberia	A34932160	TcVI	MT365386
IBA-A34932160-TcVI-H2	Iberia	A34932160	TcVI	MT365378
IBA-A34932160-TcVI-H3	Iberia	A34932160	TcVI	MT365388
IBA-A34932160-TcVI-H4	Iberia	A34932160	TcVI	MT365383
IBA-A34932160-TcVI-H5	Iberia	A34932160	TcVI	MT365380
IBA-A34932160-TcVI-H6	Iberia	A34932160	TcVI	MT365384
IBA-A34932160-TcVI-H7	Iberia	A34932160	TcVI	MT365377
IBA-A34932160-TcVI-H8	Iberia	A34932160	TcVI	MT365381
IBV-A34297450-TcI-H1	Iberville	A34297450	TcI	MT365273
IBV-A34495420-TcI-H1	Iberville	A34495420	TcI	MT365298

IBV-A34495420-TcI-H2	Iberville	A34495420	TcI	MT365253
IBV-A34495420-TcI-H3	Iberville	A34495420	TcI	MT365275
IBV-A34495420-TcI-H4	Iberville	A34495420	TcI	MT365276
IBV-A34495420-TcI-H5	Iberville	A34495420	TcI	MT365304
IBV-A34495420-TcI-H6	Iberville	A34495420	TcI	MT365255
IBV-A34495420-TcI-H7	Iberville	A34495420	TcI	MT365254
IBV-A34495420-TcI-H8	Iberville	A34495420	TcI	MT365256
IBV-A34613162-TcI-H1	Iberville	A34613162	TcI	MT365296
IBV-A34613162-TcIV-H1	Iberville	A34613162	TcIV	MT365418
IBV-A35136969-TcI-H1	Iberville	A35136969	TcI	MT365257
IBV-A35136969-TcI-H2	Iberville	A35136969	TcI	MT365258
IBV-A35136969-TcI-H3	Iberville	A35136969	TcI	MT365259
IBV-A35136969-TcI-H4	Iberville	A35136969	TcI	MT365260
IBV-A35136969-TcII-H1	Iberville	A35136969	TcII	MT365394
IBV-A35912249-TcI-H1	Iberville	A35912249	TcI	MT365354
IBV-A35912249-TcI-H2	Iberville	A35912249	TcI	MT365330
IBV-A35912249-TcI-H3	Iberville	A35912249	TcI	MT365327
IBV-A35912249-TcIV-H1	Iberville	A35912249	TcIV	MT365424
IBV-A35912249-TcIV-H2	Iberville	A35912249	TcIV	MT365416
JAC-608-TcI-H1	Jackson	608	TcI	MT365376
JAC-608-TcI-H2	Jackson	608	TcI	MT365371
JAC-608-TcI-H3	Jackson	608	TcI	MT365372
JAC-608-TcI-H4	Jackson	608	TcI	MT365375
JAC-608-TcI-H5	Jackson	608	TcI	MT365373
JAC-608-TcI-H6	Jackson	608	TcI	MT365374
JAC-651B-TcII-H1	Jackson	651B	TcII	MT365391
JAC-651B-TcII-H3	Jackson	651B	TcII	MT365392
JAC-651B-TcII-H4	Jackson	651B	TcII	MT365393
JAC-651B-TcV-H1	Jackson	651B	TcV	MT365390
JAC-717-TcI-H1	Jackson	717	TcI	MT365299
JAC-717-TcII-H1	Jackson	717	TcII	MT365402
JAC-717-TcII-H2	Jackson	717	TcII	MT365400
JAC-717-TcII-H3	Jackson	717	TcII	MT365405
JAC-717-TcIV-H1	Jackson	717	TcIV	MT365419
JAC-717-TcVI-H1	Jackson	717	TcVI	MT365382
JAC-717-TcVI-H2	Jackson	717	TcVI	MT365379
JAC-800-TcI-H1	Jackson	800	TcI	MT365267
JAC-800-TcI-H2	Jackson	800	TcI	MT365265
JAC-800-TcI-H3	Jackson	800	TcI	MT365264
JAC-800-TcI-H4	Jackson	800	TcI	MT365331
JAC-800-TcI-H5	Jackson	800	TcI	MT365333
JAC-800-TcI-H6	Jackson	800	TcI	MT365268
JAC-800-TcI-H7	Jackson	800	TcI	MT365332

JAC-800-TcI-H8	Jackson	800	TcI	MT365266
JAC-800-TcII-H1	Jackson	800	TcII	MT365408
JAC-800-TcII-H2	Jackson	800	TcII	MT365403
JAC-800-TcII-H3	Jackson	800	TcII	MT365399
JAC-800-TcII-H4	Jackson	800	TcII	MT365406
JAC-800-TcIV-H1	Jackson	800	TcIV	MT365412
JAC-800-TcVI-H1	Jackson	800	TcVI	MT365387
JAC-800-TcVI-H2	Jackson	800	TcVI	MT365385
JAC-800-TcVI-H3	Jackson	800	TcVI	MT365389
JAC-804D-TcI-H1	Jackson	804D	TcI	MT365355
JAC-804D-TcI-H10	Jackson	804D	TcI	MT365316
JAC-804D-TcI-H2	Jackson	804D	TcI	MT365288
JAC-804D-TcI-H3	Jackson	804D	TcI	MT365285
JAC-804D-TcI-H4	Jackson	804D	TcI	MT365312
JAC-804D-TcI-H5	Jackson	804D	TcI	MT365280
JAC-804D-TcI-H6	Jackson	804D	TcI	MT365321
JAC-804D-TcI-H7	Jackson	804D	TcI	MT365318
JAC-804D-TcI-H8	Jackson	804D	TcI	MT365323
JAC-804D-TcI-H9	Jackson	804D	TcI	MT365278
NO-34630215-TcI-H1	Orleans	34630215	TcI	MT365325
NO-34630215-TcI-H2	Orleans	34630215	TcI	MT365369
NO-34839412Q_TcI-H1	Orleans	34839412Q	TcI	MT365357
NO-34839412Q_TcI-H2	Orleans	34839412Q	TcI	MT365343
NO-34839412Q_TcI-H3	Orleans	34839412Q	TcI	MT365344
NO-34839412Q_TcI-H4	Orleans	34839412Q	TcI	MT365346
NO-3503251-TcI-H1	Orleans	3503251	TcI	MT365289
NO-3503251-TcI-H2	Orleans	3503251	TcI	MT365281
NO-3503251-TcI-H3	Orleans	3503251	TcI	MT365313
NO-3503251-TcI-H4	Orleans	3503251	TcI	MT365356
NO-3503251-TcI-H5	Orleans	3503251	TcI	MT365279
NO-3503251-TcI-H6	Orleans	3503251	TcI	MT365322
NO-3503251-TcI-H7	Orleans	3503251	TcI	MT365286
NO-3503251-TcI-H8	Orleans	3503251	TcI	MT365324
NO-35156400-TcI-H1	Orleans	35156400	TcI	MT365352
SMA-A34637414-TcI-H1	Saint Martin	A34637414	TcI	MT365366
SMA-A34637414-TcI-H2	Saint Martin	A34637414	TcI	MT365363
SMA-A34637414-TcI-H3	Saint Martin	A34637414	TcI	MT365365
SMA-A35705312-TcI-H1	Saint Martin	A35705312	TcI	MT365360
SMA-A35705312-TcIV-H1	Saint Martin	A35705312	TcIV	MT365417
SMV-A34564762-TcI-H1	Saint Martinville	A34564762	TcI	MT365341
SMV-A34564762-TcI-H2	Saint Martinville	A34564762	TcI	MT365339
TAN-53874-TcI-H1	Tangipahoa	53874	TcI	MT365358

TAN-53874-TcIV-H1	Tangipahoa	53874	TcIV	MT365409
TAN-53874-TcIV-H2	Tangipahoa	53874	TcIV	MT365410
TAN-56694-TcI-H1	Tangipahoa	53874	TcI	MT365284
TAN-56694-TcI-H2	Tangipahoa	53874	TcI	MT365361
TAN-56694-TcIV-H1	Tangipahoa	53874	TcIV	MT365413
WAL-D2016159-TcI-H1	Walker	D2016159	TcI	MT365282
