# Supplementary material for: Genetic diversity of Trypanosoma cruzi parasites infecting dogs in southern Louisiana sheds light on parasite transmission cycles and serological diagnostic performance
Source: PLoS Negl Trop Dis. 2020 Dec 17;14(12):e0008932. doi: 10.1371/journal.pntd.0008932 (PMC7775123; doi:10.1371/journal.pntd.0008932)
Supplement: S2 Table — (PDF) [file pntd.0008932.s002.pdf]

**Supplementary Table S2. List of sequences from vectors and other hosts**

| Host/vector | Locality | Parish | Accession # |
|---|---|---|---|
| Mouse | New Orleans | Orleans | MH316617 |
| Mouse | New Orleans | Orleans | MH316618 |
| Mouse | New Orleans | Orleans | MH316619 |
| Mouse | New Orleans | Orleans | MH316620 |
| Mouse | New Orleans | Orleans | MH316624 |
| Mouse | New Orleans | Orleans | MH316626 |
| Mouse | New Orleans | Orleans | MH316627 |
| Mouse | New Orleans | Orleans | MH316628 |
| Mouse | New Orleans | Orleans | MH316629 |
| Mouse | New Orleans | Orleans | MH316630 |
| Mouse | New Orleans | Orleans | MH316632 |
| Mouse | New Orleans | Orleans | MH316634 |
| Mouse | New Orleans | Orleans | MH316635 |
| Mouse | New Orleans | Orleans | MH316636 |
| Mouse | New Orleans | Orleans | MH316637 |
| Mouse | New Orleans | Orleans | MH316638 |
| Mouse | New Orleans | Orleans | MH316641 |
| Mouse | New Orleans | Orleans | MH316642 |
| Mouse | New Orleans | Orleans | MH316663 |
| Mouse | New Orleans | Orleans | MH316668 |
| Mouse | New Orleans | Orleans | MH316671 |
| Mouse | New Orleans | Orleans | MH316692 |
| Mouse | New Orleans | Orleans | MH316701 |
| Mouse | New Orleans | Orleans | MH316708 |
| Mouse | New Orleans | Orleans | MH316709 |
| Mouse | New Orleans | Orleans | MH316710 |
| Mouse | New Orleans | Orleans | MH316716 |
| Mouse | New Orleans | Orleans | MH316717 |
| Mouse | New Orleans | Orleans | MH316739 |
| Mouse | New Orleans | Orleans | MH316741 |
| Mouse | New Orleans | Orleans | MH316742 |
| Mouse | New Orleans | Orleans | MH316746 |
| Mouse | New Orleans | Orleans | MH316747 |
| Mouse | New Orleans | Orleans | MH316748 |
| Mouse | New Orleans | Orleans | MH316750 |
| Mouse | New Orleans | Orleans | MH316751 |
| Mouse | New Orleans | Orleans | MH316753 |
| NHP | Covington | Tammany | MH629765 |

| | | | |
|---|---|---|---|
| NHP | Covington | Tammany | MH629771 |
| NHP | Covington | Tammany | MH629772 |
| NHP | Covington | Tammany | MH629777 |
| NHP | Covington | Tammany | MH629778 |
| NHP | Covington | Tammany | MH629779 |
| NHP | Covington | Tammany | MH629782 |
| NHP | Covington | Tammany | MH629783 |
| NHP | Covington | Tammany | MH629783 |
| NHP | Covington | Tammany | MH629803 |
| NHP | Covington | Tammany | MH629804 |
| NHP | Covington | Tammany | MH629805 |
| NHP | Covington | Tammany | MH629811 |
| NHP | Covington | Tammany | MH629815 |
| NHP | Covington | Tammany | MH629817 |
| NHP | Covington | Tammany | MH629837 |
| NHP | Covington | Tammany | MH629838 |
| NHP | Covington | Tammany | MH629839 |
| NHP | Covington | Tammany | MH629840 |
| Rat | New Orleans | Orleans | MH316625 |
| Rat | New Orleans | Orleans | MH316633 |
| Rat | New Orleans | Orleans | MH316659 |
| Rat | New Orleans | Orleans | MH316672 |
| Rat | New Orleans | Orleans | MH316691 |
| Rat | New Orleans | Orleans | MH316703 |
| Rat | New Orleans | Orleans | MH316718 |
| *T. sanguisuga* | Belle Chasse | Plaquemines | MN908438 |
| *T. sanguisuga* | Lacombe | St. Tammany | MN908412 |
| *T. sanguisuga* | Lacombe | St. Tammany | MN908436 |
| *T. sanguisuga* | Lacombe | St. Tammany | MN908439 |
| *T. sanguisuga* | Lacombe | St. Tammany | MN908440 |
| *T. sanguisuga* | Lacombe | St. Tammany | MN908441 |
| *T. sanguisuga* | Lacombe | St. Tammany | MN908442 |
| *T. sanguisuga* | LaPlace | St. John | MN908390 |
| *T. sanguisuga* | LaPlace | St. John | MN908390 |
| *T. sanguisuga* | LaPlace | St. John | MN908401 |
| *T. sanguisuga* | LaPlace | St. John | MN908402 |
| *T. sanguisuga* | LaPlace | St. John | MN908403 |
| *T. sanguisuga* | LaPlace | St. John | MN908404 |
| *T. sanguisuga* | LaPlace | St. John | MN908405 |
| *T. sanguisuga* | LaPlace | St. John | MN908406 |
| *T. sanguisuga* | LaPlace | St. John | MN908407 |
| *T. sanguisuga* | LaPlace | St. John | MN908408 |
| *T. sanguisuga* | LaPlace | St. John | MN908409 |

| | | | |
|---|---|---|---|
| *T. sanguisuga* | LaPlace | St. John | MN908410 |
| *T. sanguisuga* | LaPlace | St. John | MN908411 |
| *T. sanguisuga* | LaPlace | St. John | MN908413 |
| *T. sanguisuga* | LaPlace | St. John | MN908422 |
| *T. sanguisuga* | LaPlace | St. John | MN908422 |
| *T. sanguisuga* | Livingston | Livingston | MN908382 |
| *T. sanguisuga* | New Orleans | Orleans | MN908383 |
| *T. sanguisuga* | New Orleans | Orleans | MN908384 |
| *T. sanguisuga* | New Orleans | Orleans | MN908385 |
| *T. sanguisuga* | New Orleans | Orleans | MN908387 |
| *T. sanguisuga* | New Orleans | Orleans | MN908388 |
| *T. sanguisuga* | New Orleans | Orleans | MN908389 |
| *T. sanguisuga* | New Orleans | Orleans | MN908391 |
| *T. sanguisuga* | New Orleans | Orleans | MN908392 |
| *T. sanguisuga* | New Orleans | Orleans | MN908431 |
| *T. sanguisuga* | New Orleans | Orleans | MN908434 |
| *T. sanguisuga* | New Orleans | Orleans | MN908435 |
| *T. sanguisuga* | New Orleans | Orleans | MN908394 |
| *T. sanguisuga* | New Orleans | Orleans | MN908397 |
| *T. sanguisuga* | New Orleans | Orleans | MN908398 |
| *T. sanguisuga* | New Orleans | Orleans | MN908398 |
| *T. sanguisuga* | New Orleans | Orleans | MN908400 |
| *T. sanguisuga* | Sorrento | Ascension | MN908414 |
| *T. sanguisuga* | Sorrento | Ascension | MN908414 |
| *T. sanguisuga* | Sorrento | Ascension | MN908415 |
| *T. sanguisuga* | Sorrento | Ascension | MN908416 |
| *T. sanguisuga* | Sorrento | Ascension | MN908419 |
| *T. sanguisuga* | St. Martinville | St. Martin | MN908393 |
| *T. sanguisuga* | St. Martinville | St. Martin | MN908395 |
| *T. sanguisuga* | Violet | St. Bernard | MN908401 |

NHP: non-human primate
