# Supplementary figures and images for: Genetic diversity of Trypanosoma cruzi parasites infecting dogs in southern Louisiana sheds light on parasite transmission cycles and serological diagnostic performance
Source: PLoS Negl Trop Dis. 2020 Dec 17;14(12):e0008932. doi: 10.1371/journal.pntd.0008932 (PMC7775123; doi:10.1371/journal.pntd.0008932)

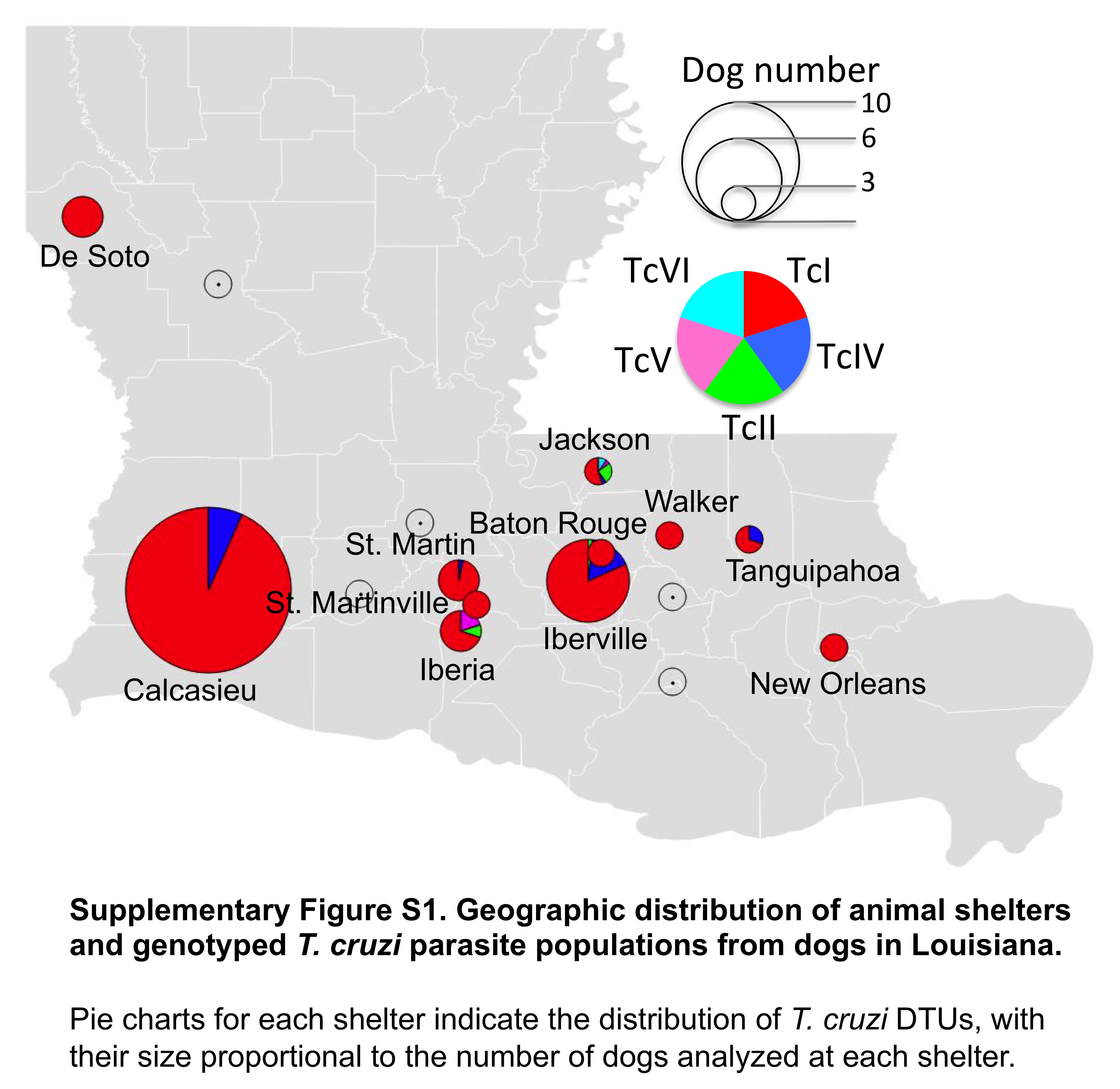

Supplement: S1 Fig — Pies charts for each shelter indicate the distribution of T. cruzi DTUs, with their size proportional to the number of dogs analyzed at each shelter. (TIF) [file pntd.0008932.s003.tif]

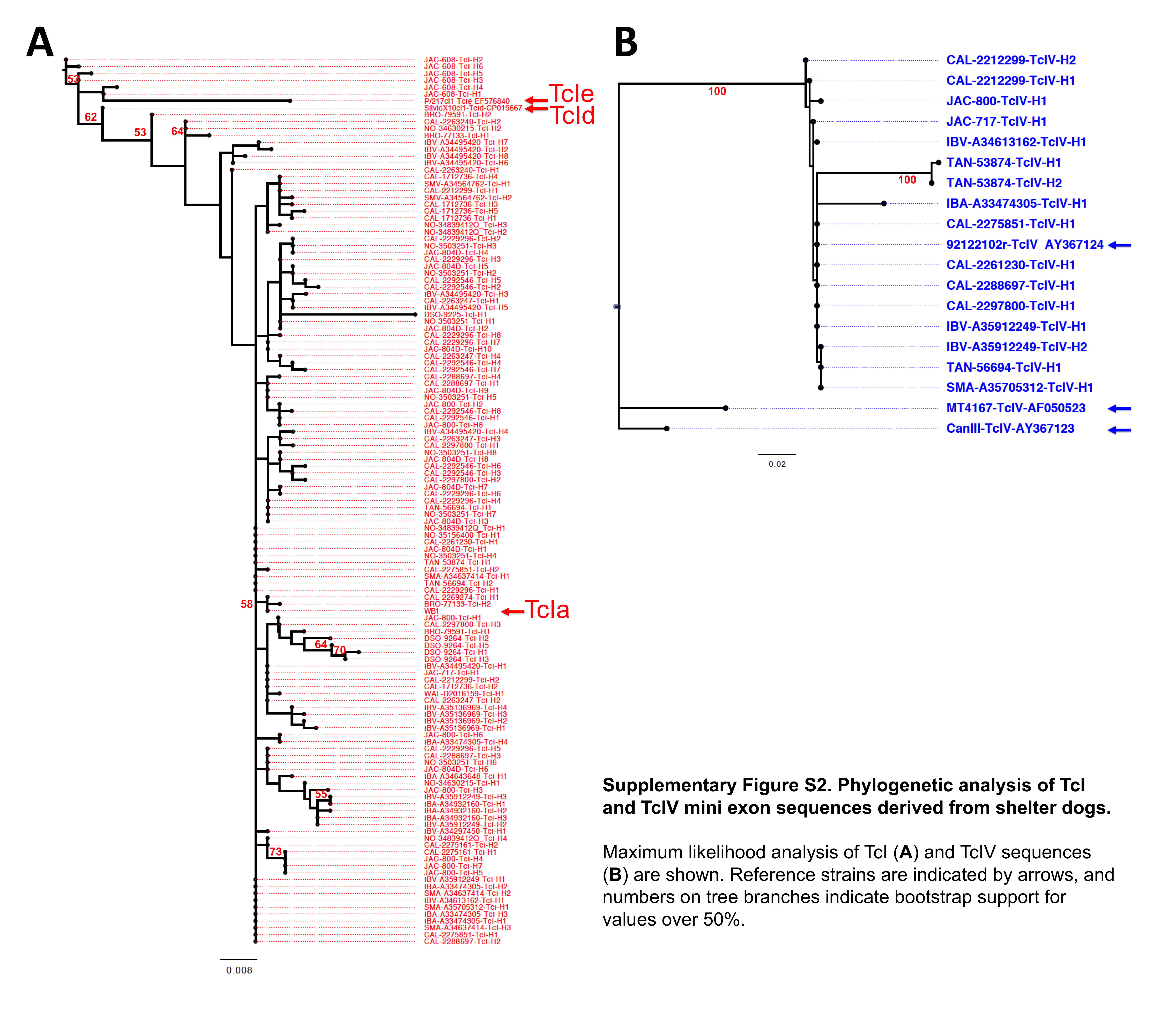

Supplement: S2 Fig — Maximum likelihood analysis of TcI (A) and TcIV sequences (B) is shown. Reference strains are indicated by arrows, and numbers on branches indicate bootstrap support for values over 50%. (TIF) [file pntd.0008932.s004.tif]
