# Supplementary material for: Genetic diversity of Trypanosoma cruzi parasites infecting dogs in southern Louisiana sheds light on parasite transmission cycles and serological diagnostic performance
Source: PLoS Negl Trop Dis. 2020 Dec 17;14(12):e0008932. doi: 10.1371/journal.pntd.0008932 (PMC7775123; doi:10.1371/journal.pntd.0008932)
Supplement: S3 Fig — TCS networks were constructed based on mini-exon sequences from dogs, mice, rats, non-human primates (NHP) and Triatoma sanguisuga from southern Louisiana. Nodes represent haplotypes, with their size proportional to the number of sequences, and they are color-coded by species. Ticks on branches indicate the number of mutations from one haplotype to the next. (A) TcI DTU. Arrows point to reference sequences from strains Raccoon70 (TcIa) and SylvioX10 (TcId). (B) TcII, TcV and TcVI DTUs. Arrows point to reference sequences from strains Tu18 (TcII), SC43 (TcV), and CL (TcVI). (PDF) [file pntd.0008932.s005.pdf]

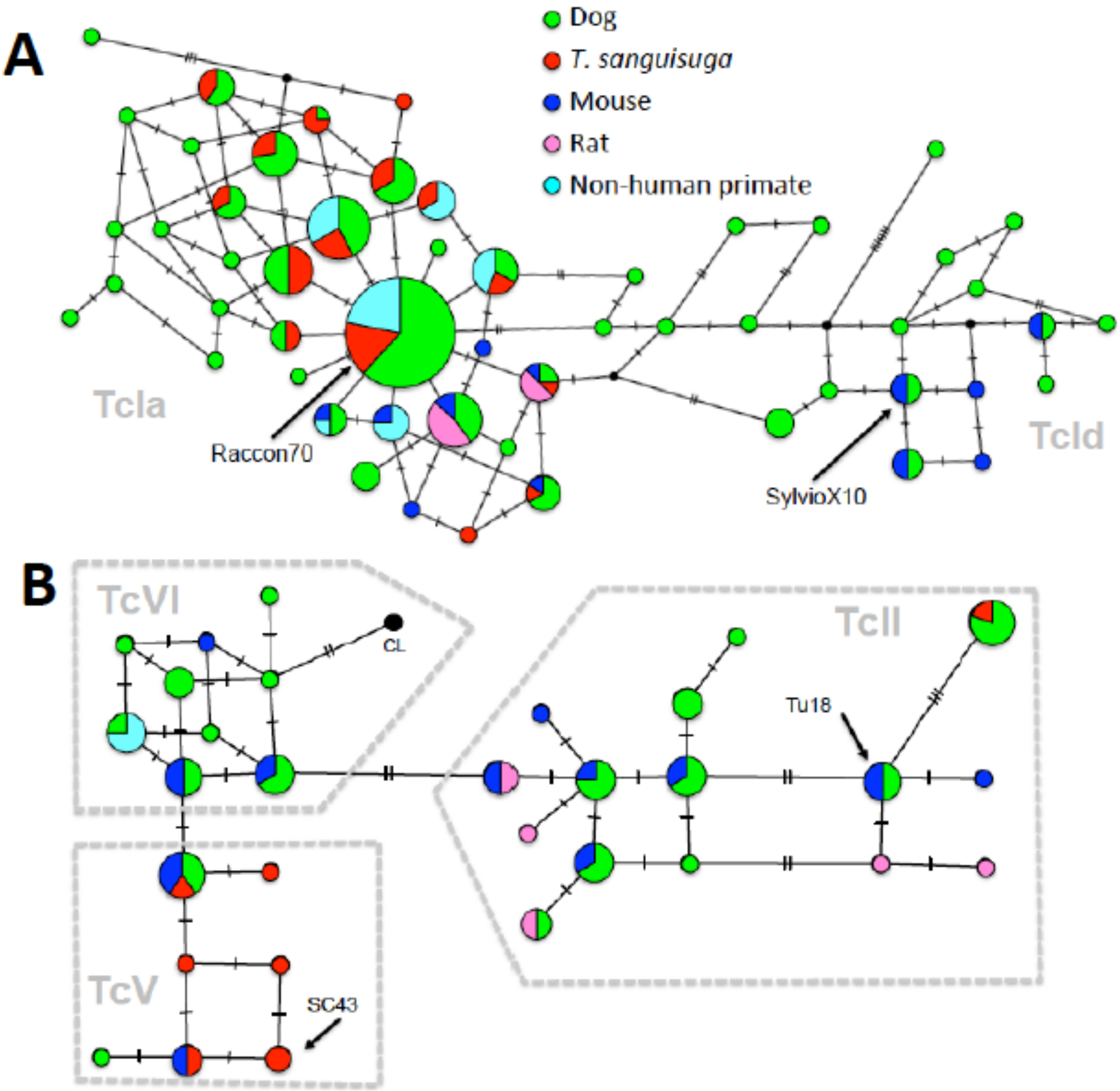

Supplementary Figure S3. Mini-exon haplotype networks from mammalian hosts and triatomine vectors from southern Louisiana.

TCS networks were constructed based on mini-exon sequences from dogs, mice, rats, non-human primates (NHP) and *Triatoma sanguisuga* from southern Louisiana. Nodes represent haplotypes, with their size proportional to the number of sequences, and they are color-coded by species. Ticks on branches indicate the number of mutations from one haplotype to the next. (**A**) TcI DTU. Arrows point to reference sequences from strains Raccoon70 (TcIa) and SylvioX10 (TcId). (**B**) TcII, TcV and TcVI DTUs. Arrows point to reference sequences from strains Tu18 (TcII), SC43 (TcV), and CL (TcVI).

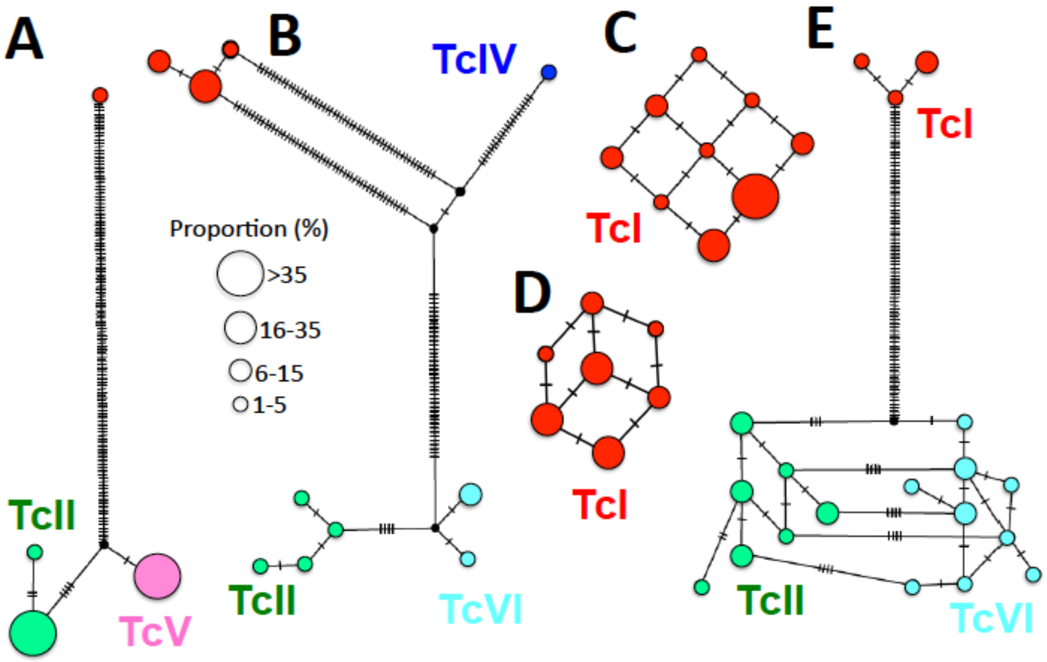

Supplementary Figure S4. Examples of mini-exon haplotype networks from individual dogs.
TCS networks were constructed based on mini-exon sequences from individual dogs in each panel (**A, B, C, D, E**). Nodes represent haplotypes, with their size proportional to their proportion as indicated, and they are color-coded by DTUs. Ticks on branches indicate the number of mutations from one haplotype to the next.
