# Supplementary material for: Genetic diversity of Trypanosoma cruzi parasites infecting dogs in southern Louisiana sheds light on parasite transmission cycles and serological diagnostic performance
Source: PLoS Negl Trop Dis. 2020 Dec 17;14(12):e0008932. doi: 10.1371/journal.pntd.0008932 (PMC7775123; doi:10.1371/journal.pntd.0008932)
Supplement: S4 Fig — TCS networks were constructed based on mini-exon sequences from individual dogs (A, B, C, D, E). Nodes represent haplotypes, with their size proportional to their proportion as indicated, and they are color-coded by DTUs. Ticks on branches indicate the number of mutations from one haplotype to the next. (PDF) [file pntd.0008932.s006.pdf]

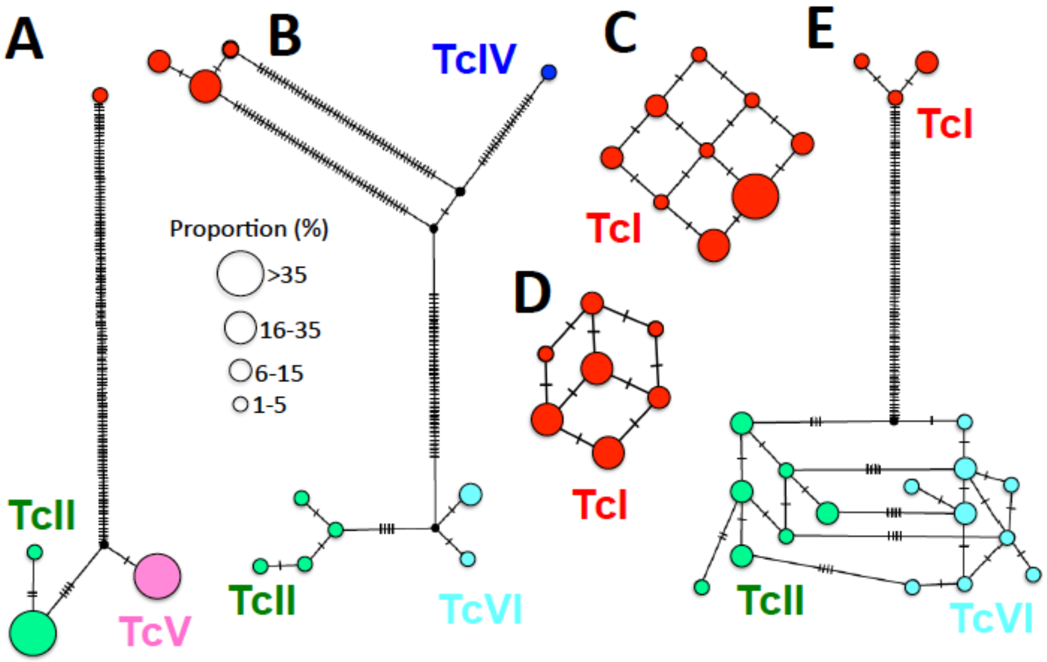

Supplementary Figure S4. Examples of mini-exon haplotype networks from individual dogs.

TCS networks were constructed based on mini-exon sequences from individual dogs in each panel (**A, B, C, D, E**). Nodes represent haplotypes, with their size proportional to their proportion as indicated, and they are color-coded by DTUs. Ticks on branches indicate the number of mutations from one haplotype to the next.
