# Supplementary material for: Genetic diversity of Trypanosoma cruzi parasites infecting dogs in southern Louisiana sheds light on parasite transmission cycles and serological diagnostic performance
Source: PLoS Negl Trop Dis. 2020 Dec 17;14(12):e0008932. doi: 10.1371/journal.pntd.0008932 (PMC7775123; doi:10.1371/journal.pntd.0008932)
Supplement: Attachment — Submitted filename: Answers to reviewers.docx [file pntd.0008932.s007.docx]

Overall, this is, potentially, an interesting contribution. However, there are important comments from the reviewers that the authors should address, particularly regarding the vector data.

**ANSWER: We thank the editors and reviewers for their comments and opportunity to improve our study. We address their comments below.**

Reviewer's Responses to Questions

<b>Key Review Criteria Required for Acceptance?</b></br></br>

As you describe the new analyses required for acceptance, please consider the following:</br></br>

<b>Methods</b></br></br>

-Are the objectives of the study clearly articulated with a clear testable hypothesis stated?</br>

-Is the study design appropriate to address the stated objectives?</br>

-Is the population clearly described and appropriate for the hypothesis being tested?</br>

-Is the sample size sufficient to ensure adequate power to address the hypothesis being tested?</br>

-Were correct statistical analysis used to support conclusions?</br>

-Are there concerns about ethical or regulatory requirements being met?</br></br>

Reviewer #1: (No Response)

Reviewer #2: All method-related aspects have been covered according to current good practice.

**ANSWER: We thank the reviewer for his/her appreciation.**

Reviewer #3: The mini-exon gene is multicopy and it is thus an odd choice for this type of work as you are dealing with multiple variable copies within a single genome as well as variation among strains in mixed infections. It would have made much more sense to use a single-copy locus that provides enough DTU resolution. In that context the description of how sequences are mapped and assembled (?) for each sample has to be expanded (p. 7). Checking the cited reference #19 there is really not enough information there to know what was done and how. No information on quality control checks, removal or chimeras and identification of paralogous copies.

**ANSWER: Many markers have been proposed for T. cruzi genotyping, but most are too conserved to present diversity within DTUs. Also, single copy loci are excellent to work with cultured parasite strains, but lack sensitivity for genotyping parasites in mammalian hosts, where parasitemia is extremely low. In this context, the mini-exon marker is well suited because it provides a good resolution of DTUs, but is also sufficiently variable to show within DTU diversity. As a multicopy sequence, it also increases the sensitivity for genotyping parasites in blood samples. Note that in spite of this sensitivity, we could successfully genotype only 40/73 PCR positive dogs.**

**As suggested, we have added methodological details for sequence mapping and assembly (Page 7, lines 159-169).**

--------------------

<b>Results</b></br></br>

-Does the analysis presented match the analysis plan?</br>

-Are the results clearly and completely presented?</br>

-Are the figures (Tables, Images) of sufficient quality for clarity?</br></br>

Reviewer #1: I know that the sequence data has been deposited in GenBank, but it would be helpful if the authors would include a supplementary table that lists the accession numbers along with the identity and location of the

dogs from which these were obtained. Otherwise, it may be difficult or impossible for a reader to determine

when two or more sequences were obtained from the same host individual.

**ANSWER: As suggested, we have added Supplementary Table S1 listing the sequences and associated metadata.**

Reviewer #2: Figures and other results are clearly  presented, no flaw detected.

**ANSWER: We thank the reviewer for his/her appreciation.**

Reviewer #3: Please provide examples of diversity within a single sample to get a sense of intragenomic diversity in the locus. You can do this with in a Supplementary figure or table.

**ANSWER: As suggested, we have added Supplementary Figure S4, to show haplotype networks from 5 dogs to illustrate sequence diversity within each animal.**

Why is the TcIII reference not shown in Figure 1? This is odd given that it is well known that TcV and TcVI are hybrids between TcII and TcIII. Are mini-exon sequences from TcV and TcVI completely different from those of TcII and TcIII or do they have a mix of haplotypes from each type as observed in any other nuclear loci? Given the hybrid nature of TcV and TcVI it is odd that you did not pick up expected TcII sequences in those samples (but in Figure 4 every case with TcV or VI shows a TcII, see comment below). How do you explain this finding?

**ANSWER: TcIII was not included in Figure 1 because no TcIII was detected in our samples. In any case, previous work has shown that TcIII mini-exon sequences are completely different from that of TcV or TcVI hybrid strains (See for example Lopez-Cancino et al, 2015; Villanueva-Lizama et al, 2019; Majeau et al, 2019; (Maiguashca Sanchez et al. 2020).**

**Regarding the co-occurrence of TcV or TcVI sequences with TcII sequences, it is indeed a possibility that these haplotypes are paralogous sequences derived from the same hybrid parasite strain rather than from strain from different DTUs. For example, we did show before that TcVI strains TCC and CL Brener harbored a small proportion of mini-exon sequences that clustered with TcII references, but these represented only 5.1 and 2.8% of their paralogous mini-exon sequences, respectively (Villanueva-Lizama et al. 2019). Thus, the observation of TcVI and TcII sequences in comparable proportions in dogs (about 40-60% of sequences for each DTU as indicated in Figure 3) is more compatible with the presence of parasite strains from multiple DTUs. These considerations have been added to the discussion for greater clarity (page 16, lines 388-392).**

Figure 2: it is not clear where the data from vectors comes from, what is the sample size, etc. There was no information in the manuscript about those samples. It is also not obvious why presenting the data in a PCA plot is better than showing again a display in a phylogenetic tree to depict more clearly the relationships between samples collected in the dogs and the vectors (from unknown precedence). Are there that many non-dog samples that showing them in a tree with select dog samples is not possible? There is so much overlap among the similar samples in the PCA that it is not possible to make sense of the meaning of the findings. For instance: the TcI cluster in part A seems to be dominated by Dog samples, but there are other samples covered by the green dog circles, what are they?

**ANSWER: As suggested, we have added additional information of the source of vector data, which has been published in a separate manuscript. We also added Supplementary Table S2, which lists all sequences from vectors and other mammalian hosts that we used for these comparisons. We selected PCA as it is very powerful to summarize large sequence data sets, allowing to easily compare sequence sets among groups, which was the purpose of this analysis. The strong overlap of data points indeed clearly shows that highly similar T. cruzi sequences are shared among the indicated hosts and vectors. We have nonetheless added Supplementary Figure S3, which shows haplotype networks of the corresponding mini-exon sequences from the different mammalian hosts and vectors. We show a network for TcI and a network for TcII, TcV and TcVI. Both networks indicate that many haplotypes from dogs were also detected in other species, or were closely related to those from other species, supporting the conclusions from the PCA analysis that T. cruzi parasites with identical/similar haplotypes are circulating in these species in the region.**

Figure 4: this is a cool figure and one that directly shows the value of next gen sequencing to determine mixed infection levels. However, why are there only 40 samples shown? In the methods you said that 73 dogs were sampled. Something interesting is that every case with TcV or TcVI has TcII. The authors interpret this as evidence of strain coocurrence but it may be just a case of the two haplotypes from hybrids being picked up in the samples.

**ANSWER: We thank the reviewer for his/her appreciation. We do mention in the results that “A total of 73 samples from *T. cruzi* PCR positive dogs were included for genotyping using the mini-exon marker, and we were able to recover 178 quality mini-exon sequences from 40 samples (55%).” This is due to the limited sensitivity of the genotyping PCR compared to diagnostic PCR, in spite of the multiple copy number of the mini-exon marker.**

**Regarding the co-occurrence of TcV or TcVI sequences with TcII sequences, it is indeed a possibility that these haplotypes are paralogous sequences derived from the same hybrid parasite strain rather than from strain from different DTUs. For example, we did show before that TcVI strains TCC and CL Brener harbored a small proportion of mini-exon sequences that clustered with TcII references, but these represented only 5.1 and 2.8% of their paralogous mini-exon sequences, respectively (Villanueva-Lizama et al. 2019). Thus, the observation of TcVI and TcII sequences in comparable proportions in dogs (about 30-60% of sequences for each DTU as indicated in Figure 3) is more compatible with the presence of parasite strains from multiple DTUs. These considerations have been added to the discussion for greater clarity (Page 16, lines 441-445).**

--------------------

<b>Conclusions</b></br></br>

-Are the conclusions supported by the data presented?</br>

-Are the limitations of analysis clearly described?</br>

-Do the authors discuss how these data can be helpful to advance our understanding of the topic under study?</br>

-Is public health relevance addressed?</br></br>

Reviewer #1: (No Response)

Reviewer #2: Results are fully discussed and conclusions supported by the data. Last paragraph of Discussion is just a repetition of said concepts and should be deleted. The study has some implications for better diagnosis of canine infections.

**ANSWER: We thank the reviewer for his/her appreciation. We agree that the past paragraph mostly restates the major points of the discussion, but we believe that it is helpful in summarizing the major take-home points of the study and prefer not to delete it.**

A major result needs more attention: Can the authors discuss potential limitations of their approach in terms of unspecific results, for example, the finding of TcV and TcVI? Did any other authors in the US (other than this research team) reported the finding of these DTUs there? Were their methods limited in this respect? Any suggestion on the source of these DTUs (e.g., humans?

**ANSWER: We agree with the reviewer that this is an important point and would argue that genotyping by NGS has an increased sensitivity to detect infections with T. cruzi parasites from multiple DTUs, which are often lost when genotyping by PCR only or even using Sanger sequencing of PCR products, as we have discussed before (Majeau et al. 2019). We hope other research groups will become interested in testing this approach in other regions in the US and elsewhere. The presence of TcII, TcV and TcVI in the US is likely not new nor coming from humans, and additional phylogenetic studies with multiple markers should help clarify their origin. Some of these considerations have been added to the discussion (page 15, lines 432-435).**

Reviewer #3: A major conclusion is the finding of a connection between genetic diversity in TcI infections and performance of serological tests, which seems very interesting and important. I just wonder how relevant is it given the small sample sizes. In Fig 5B the seronegatives cover most of the PCA plot and have complete overlap with the seropositives.

**ANSWER: We believe that this is a very relevant point and that in spite of its limitations, our sample size is one of the largest available so far. As we mentioned, our data indicate that genetic differentiation based on the mini-exon marker may not strictly reflect the antigenic differentiation of the strains (Page 14, lines 359-360), but is nonetheless able to support the hypothesis that parasite genetic diversity is a key factor affecting serological test performance. Studies aimed at characterizing the antigenic determinants of parasite strains may reveal larger differences among strains.**

--------------------

<b>Editorial and Data Presentation Modifications?</b></br><br/>

Use  this section for editorial suggestions as well as relatively minor modifications of existing data that would enhance clarity. If the only modifications needed are minor and/or editorial, you may wish to recommend “Minor Revision” or “Accept”.

Reviewer #1: There are several sentences containing minor grammatical errors or confusing wording. Suggested corrections are listed below:

line 62: differential selection of lineages.

line 163: was assessed

line 176: was assessed

line 182: In total, 73 samples ... were

line 265: Overall, TcI parasites

line 327: ticks on branches

line 354: point to a high diversity

line 363: implication of such infections

line 368: while the host may better control others.

line 382: and DTUs may partition among host species

line 385: for serological diagnostics.

line 394: two of which were based on a

line 397: the genetic distance of each sequence to that of the local

**ANSWER: These errors and typos have been corrected as suggested.**

Reviewer #2: check a few typos, e.g. line 363

**ANSWER: These typos have been corrected as suggested.**

Reviewer #3: Sentence in line 78: provide reference

**ANSWER: We have added references as suggested.**

--------------------

<b>Summary and General Comments</b></br></br>

Use  this section to provide overall comments, discuss strengths/weaknesses of the study, novelty, significance, general execution and scholarship. You may also include additional comments for the author, including concerns about dual publication, research ethics, or publication ethics. If requesting major revision, please articulate the new experiments that are needed.

Reviewer #1: This manuscript describes genetic variation at a nuclear barcoding marker in Trypanosoma cruzi parasites

infecting domestic dogs in animal shelters in southern Louisiana. To resolve mixed infections, the

investigators used PCR amplification of the mini-exon marker followed by deep sequencing on a MiSeq platform. This approach yielded genetic data for 39 out of 73 PCR-positive animals. Analysis of this data

revealed the following points:

1) Mixed infections (as evidenced by the mini-exon locus) are common in dogs in this region.

2) Genetic variation in canine infections is high in this region, with the authors detecting several sequence

types (DTU's) for the first time within the US.

3) North American isolates are genetically distinct from South American reference strains.

4) The distribution of mini-exon genotypes differs between the domestic dogs sampled in this study and

Triatomine vectors investigated in an earlier study.

5) Variation in the mini-exon sequence is significantly correlated with serological test performance,

with certain mini-exon sequences being associated with negative or discordant test results more frequently

than expected by chance.

On the whole, this is an solidly-designed study which makes an interesting contribution to our

understanding of canine trypanosomiasis in the southeastern US. However, I have one major criticism, pertinent to result #4. The authors perform a chi-squared test comparing the frequencies of mini-exon haplotypes isolated from dogs with those isolated in vectors and find that these distributions are significantly different. They then conclude that "these data strongly suggest the occurrence of strain/DTU differential selection in vectors and hosts". While this conclusion may be correct, I believe that it is unwarranted by the present study for two reasons.

One difficulty is with the validity of the chi-squared test for frequency data sampled in structured populations. A core assumption of this test is that the samples are independent of one another. However,

in the present study, multiple dogs have been sampled from the same shelter and, by virtue of their geographical proximity, arguably are more likely to share related parasite genotypes than dogs sampled from distant shelters. Although the authors do not explicitly test for geographical structure in their data, Figure S1 demonstrates that there is geographical variation in the haplotype distribution within shelter dogs. Likewise, because most infections are mixed, many of the samples used to calculate the haplotype

frequencies will have been collected from the same host. However, this too may lead to violations of the

independence assumption if either drift or selection is occurring within individual hosts. For these reasons,

I am skeptical of the significance of this test result. As an aside, it may be of interest to explicitly test

for geographical structure in this data, noting that there are two levels of organization: sequences within

hosts and hosts within structures, e.g., use a hierarchical AMOVA.

A potentially more serious objection comes from the fact that we are not told anything about the

study design used to generate the vector data, which is from an unpublished manuscript. Unless the

hosts and the vectors were sampled at the same time and in the same locations using a balanced sample design, we cannot exclude the possibility that differences between the samples are due to confounding

spatial or temporal variation in the genetic composition of the parasite population. This effect will be

exacerbated if there are clonal expansions or non-random interactions between the hosts and vectors.

I believe that the authors should take two steps to address these concerns. First, they should be much

more cautious when interpreting these results and note both the statistical and interpretive challenges

that the data presents. Secondly, it would be helpful for them to include some information concerning

when and where the vectors were sampled.

**ANSWER: We thank the reviewer for his/her detailed analysis, and agree that we may have reached a hasty interpretation regarding potential differences in DTU frequencies between dogs and vectors. The caveats pointed out by the reviewer are certainly valid, as well as the suggestions for a stronger analysis. However, we recognize that the study was not designed to directly address this question and perform a rigorous analysis of geographic structuration of the parasite and we only intended to provide a general comparison of strains circulating in the region. Indeed, sample size per location is likely too low for a reliable evaluation of spatial distribution as noted (Page 10, lines 282-283). We have thus reworded our interpretation of these data and attempted to be much more cautious by discussing these limitations (Pages 16-17, lines 451-477). We also modified the abstract and author summary accordingly. We have also added additional information on the sequence data from vectors, which have now been published in a separate manuscript, and we provide Supplementary Table S2, which lists the sequences from vector and other mammalian hosts used in the analysis.**

Reviewer #2: Great work, very well written and presented, some very surprising results (at least for me), further studies for confirmation needed.

**ANSWER: We appreciate the reviewer’s comments and certainly agree that additional studies should help clarify further the diversity of *T. cruzi* circulating in the region.**

Reviewer #3: See comments above for all suggestions

**ANSWER: We hope that we have addressed the reviewer’s suggestions above.**

**References:**

**Lopez-Cancino, S. A., E. Tun-Ku, H. K. Felix, C. N. Ibarra-Cerdena, A. Izeta-Alberdi, A. Pech-May, C. J. Mazariegos-Hidalgo, A. Valdez-Tah and J. M. Ramsey. 2015. Landscape ecology of *Trypanosoma cruzi* in the southern Yucatan Peninsula. Acta Trop 151: 58-72.**

**Maiguashca Sanchez, J., S. O. B. Sueto, P. Schwabl, M. J. Grijalva, M. S. Llewellyn and J. A. Costales (2020). Remarkable genetic diversity of Trypanosoma cruzi and Trypanosoma rangeli in two localities of southern Ecuador identified via deep sequencing of mini-exon gene amplicons. Parasit Vectors 13(1): 252.**

**Majeau, A., C. Herrera and E. Dumonteil (2019). An improved approach to *Trypanosoma cruzi* molecular genotyping by next-generation sequencing of the mini-exon gene. Methods Mol Biol 1955: 47-60.**

**Villanueva-Lizama, L., C. Teh-Poot, A. Majeau, C. Herrera and E. Dumonteil (2019). Molecular genotyping of *Trypanosoma cruzi* by next-generation sequencing of the mini-exon gene reveals infections with multiple parasite DTUs in Chagasic patients from Yucatan, Mexico. J Inf Dis 219(12): 1980-1988.**
