# Supplementary material for: Genetic diversity of Trypanosoma cruzi parasites infecting dogs in southern Louisiana sheds light on parasite transmission cycles and serological diagnostic performance
Source: PLoS Negl Trop Dis. 2020 Dec 17;14(12):e0008932. doi: 10.1371/journal.pntd.0008932 (PMC7775123; doi:10.1371/journal.pntd.0008932)
Supplement: Attachment — Submitted filename: Answers to reviewers comments R2.docx [file pntd.0008932.s008.docx]

Reviewer's Responses to Questions

<b>Key Review Criteria Required for Acceptance?</b></br></br>

As you describe the new analyses required for acceptance, please consider the following:</br></br>

<b>Methods</b></br></br>

-Are the objectives of the study clearly articulated with a clear testable hypothesis stated?</br>

-Is the study design appropriate to address the stated objectives?</br>

-Is the population clearly described and appropriate for the hypothesis being tested?</br>

-Is the sample size sufficient to ensure adequate power to address the hypothesis being tested?</br>

-Were correct statistical analysis used to support conclusions?</br>

-Are there concerns about ethical or regulatory requirements being met?</br></br>

Reviewer #1: (No Response)

Reviewer #2: I found the responses to other reviewers' queries appropriate.

Since one of the conclusions of this paper is related to the mismatch between serodiagnosis and parasite genetic makeup, the authors should provide more details on serodiagnostic methods, cut-off values, sensitivity and specificity for each of them, and not just provide the reference for a previous work. More in Results below.

PERMANOVA: software used?

**ANSWER: As suggested, we have added further details on serological methods (Page 8, lines 187-192) as well as the software used for PERMANOVA analyses (Page 8, lines 182-183).**

Reviewer #3: The authors have improved the description of methods and they are now satisfactory.

**ANSWER: We are glad to have improved our study and thank the reviewer for his/her appreciation.**

--------------------

<b>Results</b></br></br>

-Does the analysis presented match the analysis plan?</br>

-Are the results clearly and completely presented?</br>

-Are the figures (Tables, Images) of sufficient quality for clarity?</br></br>

Reviewer #1: (No Response)

Reviewer #2: I found the responses to other reviewers' queries appropriate.

In reference to the association between serodiagnosis outcomes and parasite genetic makeup, the authors should provide a contingency table that summarizes these specific results. As one of the claims of this manuscript is that serodiagnosis should be improved, these relationships should be made explicit. (In this regard, consider adding a reference to similar criticisms put forward by Tarleton and Cooley, this subject goes way back and beyond canine serodiagnosis).

**ANSWER: We are glad to have improved our study and thank the reviewer for his/her appreciation. As suggested, we have added references from Tarleton’s group on the shortcomings of serological tests (Page 5 and 17), as this is indeed nothing new. On the other hand, because of the rather large number of parasite sequence haplotypes to associate with serological status of the dogs, we do not think that a contingency table would better illustrate our findings. We believe that the haplotype network and PCA analysis shown in Figure 5 better summarize these data.**

Reviewer #3: Thanks for adding Figure S4 to help provide a sense of intragenomic diversity in the miniexon locus in 5 individual dogs with different infection profiles. In the figure legend please remove the end of the first sentence (“in each panel”) because it is confusing.

**ANSWER: We are glad to have improved our study and thank the reviewer for his/her appreciation. We have now revised the figure legend as suggested.**

Thanks for the clarification about what the presence of multiple haplotypes means and why TcIII is not present in infections from hybrid DTUs TcV and TcVI. The lack of resolution to determine the exact nature of mixed infections using miniexon sequences underscore the importance of also using single copy genes that will provide better resolution even if the authors claim that low parasitemia reduces chance of positive PCRs. Designing primers for PCR products of similar size for multiple markers that can be combined for amplicon next gen sequencing should provide better resolution. Food for thought for future work.

The clarification about PCA and added figures are very useful.

**ANSWER: We are glad to have improved our study and thank the reviewer for his/her appreciation.**

--------------------

<b>Conclusions</b></br></br>

-Are the conclusions supported by the data presented?</br>

-Are the limitations of analysis clearly described?</br>

-Do the authors discuss how these data can be helpful to advance our understanding of the topic under study?</br>

-Is public health relevance addressed?</br></br>

Reviewer #1: (No Response)

Reviewer #2: I found the responses to other reviewers' queries appropriate, and limitations clearly described

**ANSWER: We are glad to have improved our study and thank the reviewer for his/her appreciation.**

Reviewer #3: Generally the work is well presented, conclusions well supported, and public health relevance is pretty obvious. Something to have in mind when trying to interpret negative serological tests in individuals that are infected with the parasite (based on a positive PCR) is that serological tests only detect one aspect of the immune response. T cell responses are as important or more than antibody production but they are much harder to measure. It is possible that most of the immune response against the parasites or against some of the DTUs is driven by T cells. Some mention of this in the discussion or conclusions would be useful.

**ANSWER: This is a good point and we now mention it in the discussion (Page 17, lines 434-436).**

--------------------

<b>Editorial and Data Presentation Modifications?</b></br><br/>

Use  this section for editorial suggestions as well as relatively minor modifications of existing data that would enhance clarity. If the only modifications needed are minor and/or editorial, you may wish to recommend “Minor Revision” or “Accept”.

Reviewer #1: (No Response)

Reviewer #2: No suggestion on these items.

Reviewer #3: (No Response)

--------------------

<b>Summary and General Comments</b></br></br>

Use  this section to provide overall comments, discuss strengths/weaknesses of the study, novelty, significance, general execution and scholarship. You may also include additional comments for the author, including concerns about dual publication, research ethics, or publication ethics. If requesting major revision, please articulate the new experiments that are needed.

Reviewer #1: The authors have adequately addressed the concerns raised in my initial review of their manuscript. Apart from a few minor changes in wording (suggested below), I think that the revised manuscript is suitable for publication.

Suggested minor corrections:

p. 41: However, TcIV and TcV were sampled more frequently in vectors, while TcII and TcVI were sampled more frequently in dogs.

p. 188: The list of sequences ...

p. 307: ... were detected more frequently in vectors, while ...

p. 382: ... key to understanding parasite transmission cycles, and assessing the ...

p. 446: Expanding these observations to larger samples from additional locations and host species would ...

p. 463: ... to that of the local strain used for diagnostics ...

**ANSWER: All edits have been made as suggested.**

Reviewer #2: Interesting paper. It may stimulate others to reevaluate the occurrence of parasite DTUs in the USA.

**ANSWER: We are glad to have improved our study and thank the reviewer for his/her appreciation.**

Reviewer #3: See comments above and my first review
